# Immunopathobiology of chronic lung disease

**DOI:** 10.1002/cti2.1170

**Published:** 2020-08-24

**Authors:** Cecilia M Prêle, Gerard F Hoyne

**Affiliations:** ^1^ Centre for Respiratory Health University of Western Australia Nedlands WA 6009 Australia; ^2^ Centre for Cell Therapy and Regenerative Medicine School of Biomedical Sciences University of Western Australia Nedlands WA 6009 Australia; ^3^ Ear Science Institute Australia Nedlands WA 6009 Australia; ^4^ School of Health Sciences University of Notre Dame Australia Fremantle WA 6559 Australia

The lung epithelium is a cellular barrier that protects the lung from environmental exposure to pathogens and chemical insults that could otherwise compromise its physiological role in gas exchange during respiration. In the distal lung, the exchange of oxygen and carbon dioxide occurs within the alveoli, facilitated by type I alveolar epithelial cells (AECs). In addition to their important role in gas exchange, mucus and surfactant secreted by AECs contain a range of host‐defence proteins which aid epithelial barrier function and protection from invading pathogens. The lung epithelium is supported by a network of mesenchymal cells including fibroblasts that form the basement membrane that acts as a further layer of protection to prevent access of microbes to the underlying tissue.

Many interstitial lung diseases (ILDs) share similar clinical symptoms and findings on radiographic or pathologic assessment often making diagnosis difficult. The accurate diagnosis of an ILD is critical for the long‐term management of the disease and can inform the choice of treatment a patient receives and their prognosis. We are beginning to appreciate the importance that immune status has on disease progression and clinical outcome. In this Special Feature of *Clinical & Translational Immunology*, the reviews include a broad range of topics examining the clinical challenges associated with the diagnosis of ILDs and the nature of the innate and adaptive immune responses elicited following tissue damage in ILDs and in lung regeneration. This series of articles explores how the immune response is modulated during lung cancer and how novel immunotherapeutic approaches are being explored to treat this disease. The use of animal models to study the pathobiology of ILDs is discussed and highlights how these models may inform novel therapeutic strategies to treat ILDs or lung cancer in humans.

The review by McLean‐Tooke and colleagues examines the challenges associated with the accurate diagnosis of ILDs in patients.^1^ There are over 200 different entities of ILDs that have been described. Many exhibit low occurrence rates within the human population which can impact on the accuracy of clinical diagnosis and the ability to study disease pathogenesis. The authors focus on two main diseases: idiopathic pulmonary fibrosis, (IPF) which develops late in life and has a poor prognosis of 2–5 years, and interstitial pneumonia with autoimmune features (IPAF), a feature associated with connective tissue disorders (CTD) that include rheumatoid arthritis, systemic lupus erythematosus and systemic sclerosis. Patients who do not meet the specific criteria of CTD are given a diagnosis of IPAF. Both IPF and IPAF share similar disease symptoms, as well as radiologic and pathologic features, making clinical diagnosis difficult. Some IPAF patients can progress to become CTD, while others do not. The review examines the various diagnostic criteria of histopathology and autoimmune serology to screen for various autoantibodies to systemic autoantigens. Although previous genome‐wide association studies have identified a number of genes that influence susceptibility to familial IPF and IPAF, including *MUC5B*, *SPC* (epithelial cells), *TOLLIP* (innate immune cells) or *PARN*, *TERT* (telomere genes),^2^, ^3^, ^4^ genetic screening of IPF or IPAF for diagnostic purposes is not yet routine.

The reviews by Warheit‐Niemi *et al*.^5^ and Denneney *et al*.^6^ examine innate immune function in chronic lung disease. The discovery of pattern recognition receptors (PRRs) and their ability to bind both pathogen‐associated molecular patterns (PAMPs) and danger‐associated molecular patterns (DAMPs) have helped our understanding of the intimate connection between the innate and adaptive immune system.^7^ Triggering of PRRs on epithelial cells or innate cells such as macrophages and neutrophils stimulates the release of proinflammatory cytokines/chemokines and effector responses (phagocytosis) to help control infection and promote tissue repair mechanisms to limit inflammation. Warheit‐Niemi *et al*.^5^ provide a comprehensive overview of the innate immune response to lung immunity and fibrosis. They discuss various preclinical models in mice that have been used to dissect the effector response in lung fibrosis. Consideration is also given to the role of microbial infection as a driver of lung fibrosis. As epithelial cells, fibroblasts and innate immune cells share the ability to express PRRs, they play a pivotal role in directing the nature and chronicity of the inflammatory response. Therefore, it is crucial to understand how recognition of common respiratory pathogens or commensal organisms within the lung microbiota may impact on lung fibrosis. Denenney *et al*.^6^ focus on the role of mucins and their receptors in chronic lung disease. Mucus secreted by epithelial cells forms a natural barrier to protect the surface of the lung epithelium from microbes. The mucus is composed of a range of mucin proteins, and these can have important immunomodulatory effects on the innate and adaptive immune responses. The functions of various mucins are examined in both health and disease with a focus on pulmonary fibrosis and other chronic lung conditions such as COPD, asthma, bronchiectasis and lung cancer.

Lucas *et al*.^8^ provide an overview of the cellular and molecular mechanisms that underpin regenerative processes in the lung following acute or chronic damage. They provide insights into the role of innate and adaptive immune responses in this process. The early response to lung damage involves recognition of PAMPS or DAMPs by PRRs on tissue‐resident innate or memory T cells. The balance of TH1/TH17 and Tregs appears crucial for tissue repair processes mediated via interleukin (IL)‐17, IL‐23, IL‐10 and IL‐22. Dysregulated immune responses are the hallmark of chronic inflammatory diseases such as COPD and IPF. Lucas *et al*. describe a range of preclinical mouse studies using various anti‐cytokine therapy approaches and cytokine gene knockout models. Although some of these approaches have shown promise, this success has not yet been translated to human patients. Collectively, the three reviews by Warheit‐Niemi *et al*., Denneney *et al*. and Lucas *et al*. highlight the important role of innate and adaptive immune responses in tissue damage and repair mechanisms.

Miles *et al*.^9^ investigate a range of animal models that have been used to study the disease pathogenesis of human IPF. Some animals (e.g. dogs and horses) develop a spontaneous form of interstitial lung disease which resembles many of the clinical features of IPF by radiologic criteria. In contrast, mice do not normally develop spontaneous pulmonary fibrosis. Rather, the delivery of various chemical or drug insults can induce acute lung injury that develops into tissue fibrosis. Although the use of some of these animal models and their relevance to human disease has been debated over the years, it is generally accepted that they provide valuable insight into the cellular and molecular mechanisms driving the fibrogenic process. Furthermore, the mouse immune system is highly analogous to that of humans, and the ease of genetic manipulation in mice has made them a common choice to study disease pathogenesis. Genetic studies in congenic mouse strains have helped define genetic loci that predispose to pulmonary fibrosis following bleomycin treatment. Some of the genes are involved in activation of TCRγδ cells^10^ which are known to play crucial roles in mucosal immune regulation in mouse and humans.

The final review by Neeve *et al*.^11^ examines the role of T cells in the control of lung cancer. Anti‐tumor responses in the immune system require a coordinated response by both innate and adaptive immune cells. The activation of tumor‐specific CD8^+^ cytotoxic T cells is critical for eliminating cancer cells and reducing tumor burden. However, it is now known that tumor cells can subvert the immune system by expressing checkpoint ligands which bind to inhibitory receptors on T cells, rendering them ineffective for tumor surveillance and eradication. A range of checkpoint inhibitory receptors and ligands have been defined and are being evaluated in a range of clinical trials in cancer. Checkpoint therapy has revolutionised cancer therapy for melanoma, and it is being evaluated for the treatment of lung cancer and other cancer types. Early indications are that monotherapies of checkpoint inhibitors in lung cancer may not be effective in all tumors, thus prompting the use of combination therapies.^12^

Chronic diseases of the lung, such as pulmonary fibrosis or lung cancer, can disrupt the delicate tissue architecture and compromise gas exchange across alveoli. If disease pathology is not arrested, it can severely impact the quality of life and long‐term survival of the patient. We need to better understand the complex relationship between host genetics, the environment, and the host immune response, and how this shapes disease pathogenesis within the lung. Improved diagnostics will be developed with integration of new emerging technologies such as immune cell phenotyping, transcriptomics and metabolomics that may help to better stratify patients into appropriate clinical subgroups. This may have the benefit of improving the outcome of clinical trials of novel interventions that may be targeted to a specific response pathway.

## Conflict of interest

The authors declare no conflict of interest.

## Author contributions

**Cecilia Prêle:** Conceptualization; Writing‐original draft; Writing‐review & editing. **Gerard Hoyne:** Conceptualization; Writing‐original draft; Writing‐review & editing.
